# First report of *Babesia gibsoni* in Central America and survey for vector-borne infections in dogs from Nicaragua

**DOI:** 10.1186/1756-3305-7-126

**Published:** 2014-03-25

**Authors:** Lanjing Wei, Patrick Kelly, Kate Ackerson, Jilei Zhang, Heba S El-Mahallawy, Bernhard Kaltenboeck, Chengming Wang

**Affiliations:** 1 Yangzhou University College of Veterinary Medicine, Yangzhou, Jiangsu, P. R. China; 2 Jiangsu Co-innovation Center for the Prevention and Control of Important Animal Infectious Diseases and Zoonoses, Yangzhou, Jiangsu, P. R. China; 3 Ross University School of Veterinary Medicine, Basseterre, St. Kitts and Nevis; 4 Faculty of Veterinary Medicine, Suez Canal University, Ismailia Egypt; 5 Auburn University College of Veterinary Medicine, Auburn, Al, USA

**Keywords:** Nicaragua, *Rickettsia felis*, *Babesia gibsoni*/*vogeli*, *Hepatozoon canis*, *Anaplasma platys*, *Ehrlichia canis*, *Coxiella burnetii*, *Dirofilaria immitis*

## Abstract

**Background:**

Although many vector-borne diseases are important causes of morbidity and mortality in dogs in tropical areas and potential zoonoses, there is little information on these conditions in Central America.

**Methods:**

Seven qPCRs for vector-borne pathogens were performed on a Roche LightCycler PCR Instrument to investigate their prevalence in a convenience sample of whole blood samples from apparently healthy dogs in Nicaragua. Also, a qPCR targeting the canine hydroxymethylbilane synthase (HMBS) gene was used as an endogenous internal control and verified the quality and quantity of DNA in the samples was appropriate for the study.

**Results:**

We found DNA of *Rickettsia felis* (5%), *Babesia* spp. (26%), *Hepatozoon canis* (51%), *Anaplasma platys* (13%) and *Ehrlichia canis* (56%) in the 39 dogs studied. The qPCRs for *Coxiella burnetii* and *Dirofilaria immitis* were negative. Of the 30 (80%) dogs that were positive by qPCR, 12 (31%) were positive for one agent, 11 (28%) for two, 3 (8%) for three, and 4 (10%) for four agents.

**Conclusions:**

This is the first report of *B. gibsoni* in dogs from Central America and the first recording of vector-borne agents in dogs from Nicaragua. Dogs in Nicaragua are commonly infected with a variety of vector-borne pathogens, some of which may also infect people.

## Background

Vector-borne agents are important causes of morbidity and mortality in dogs worldwide [1-4]. Many cause zoonoses and the close relationship between people and their dogs facilitates human infections. Knowledge of the prevalences of canine vector-borne disease is thus important for veterinarians and their patients as well as for workers in the public health field [5]. There is only very limited data on vector-borne diseases in dogs from the seven Central American states with reports from only three countries, mainly Costa Rica [6-8], Panama [9-11] and Guatemala [12]. To provide further data on canine vector-borne diseases from the region, we investigated a convenience sample of dogs from Nicaragua and describe the findings in this report.

## Methods

### Dogs and blood samples

This study was approved by the Institutional Animal Care and Use Committee of the Yangzhou University College of Veterinary Medicine of China. Convenience samples of whole blood were collected into EDTA from 39 apparently healthy dogs presented at a neutering program operated by Volunteers for Intercultural and Definitive Adventure (VIDA, sites.google.com/site/rusvmvida) in April 2012. The dogs belonged to local people from underprivileged areas in Rivas, a city situated in Southwestern Nicaragua. Verbal permission was received from the owners of the dogs for blood to be used in the study. The whole blood samples were stored at 4°C for 2–3 days before aliquots (200 μL) were frozen at -80°C until DNA was extracted as described below.

### DNA extraction

The High-Pure PCR Template Preparation Kit (Roche Molecular Biochemicals, Indianapolis, IN, USA) was used to extract total nucleic acids from the whole blood aliquots as described previously [13]. Each extracted DNA was eluted in 200 μL elution buffer and stored at -80°C for qPCRs.

### Quantitative PCRs

Eight qPCRs, seven for tick-borne pathogens and one for the canine HMBS gene as an endogenous internal control, were performed on a Roche LightCycler 480-II PCR Instrument. The qPCRs were performed as described previously for *R. felis*[14], *Babesia* spp. [15], *Hepatozoon* spp. [16], *Anaplasma* spp. [17], *E. canis*[17], *C. burnetii*[18] and the canine HMBS gene [19]. The PCR products were verified by gel electrophoresis and nucleotide sequencing using forward and antisense primers (GenScript, Nanjing, China).

The Clustal Multiple Alignment Algorithm was used to compare the GenBank sequences of the 5.8S rRNA of *D. immitis* (AF217800,EU087700, EU182331.1, EU182328, AF217800)and the following primers and probes were designed specifically for *D. immitis* and validated: sense primer: 5′- TTCAATAACTCTAAGCGGGGGATCACCT-3′; anti-sense primer: 5′- TCTGATCGATATTGACCCTCAACCAGAC-3′; 6-FAM probe: 5′-TGCAGACGCATTGAGCACAAAGATTTC-(6-FAM)-3′; LCRed 640 probe: 5′-(LCRed-640)- AATGCACATTGCACCATCGGGTTGA-(Phosphate)-3′. The hybridization temperature of the qPCR was found to be optimal at 58°C and the thermal cycling protocol was as described previously [17]. A 172-bp nucleotide fragment representing the canine *D. immitis* 5.8S rRNA gene sequence was synthesized and inserted in the pIDTSMART cloning vector (Integrated DNA Technologies, Coralville, IA, USA) and used as the quantitative standard for the *D. immitis* qPCR. The DNA products were quantified using the PicoGreen® DNA fluorescence assay (Molecular Probes, Eugene, OR, USA) and the number of target molecules was calculated. Logarithmic dilutions of the DNA products were used to determine the sensitivity of the *D. immitis* qPCR, which was verified by gel electrophoresis and nucleotide sequencing (GenScript, Nanjing, China).

### Statistical analysis

The gene copy numbers determined by qPCRs were logarithmically transformed for statistical analysis using the Kruskal-Wallis non-parametric ANOVA.

## Results and discussion

The limited time available for the neutering program meant we were unable to carry out detailed investigations of the ticks found on the dogs. The overall impression, however, was that ticks were common and consisted mainly of *R. sanguineus* group. There was no regular use of acaricides for ticks and control was mostly by manual removal when tick burdens were high.

The qPCR for the HMBS gene was positive on all samples with 2.1 × 10^6^ ± 8.8 × 10^5^ copies/ml whole blood, indicating successful extraction of amplifiable DNA from all samples. The qPCR we developed for *D. immitis* consistently gave positive results with the quantitative standard we synthesized. Using the standard we found we could detect a single copy of the 5.8S rRNA gene in a qPCR reaction.

We obtained positive qPCR results for 5 of the 7 organisms we tested. Only *D. immitis* and *C. burnetii* gave negative qPCR results in all the dogs we examined. *D. immitis* is the agent of canine heartworm disease which is transmitted by mosquitoes and an important cause of lung and heart pathology in dogs worldwide [20]. There is only one report on the disease from Central America where just 2% of dogs studied in Costa Rica were seropositive [7], considerably less than that from the nearby USA where seroprevalences may be up to 49% [21]. Absence of animals positive by qPCR in our study could reflect the small sample of dogs we tested or represent a more generalized low level of infection. Further studies are needed to determine the true status of *D. immitis* infections in Nicaragua.

*C. burnetii* is the agent of Q fever in people, a disease reported to occur worldwide, apart from New Zealand [22]. Sources of human infections are generally domestic ruminants although cats have been implicated in some outbreaks [23] and dogs in only one [24]. There are no reports of *C. burnetii* in people or animals in Central America and we found no evidence of infection in dogs. Using PCR, however, is not a sensitive method to detect *C. burnetii* exposure in dogs or its presence in an area. In the only other survey for *C. burnetii* in dog blood which was conducted in Hungary, just 1/123 samples was PCR positive despite 25 being seropositive [25]. To determine the importance of Q fever in Central America, studies are needed on people and livestock which are the major reservoirs of infection.

**Figure 1:**
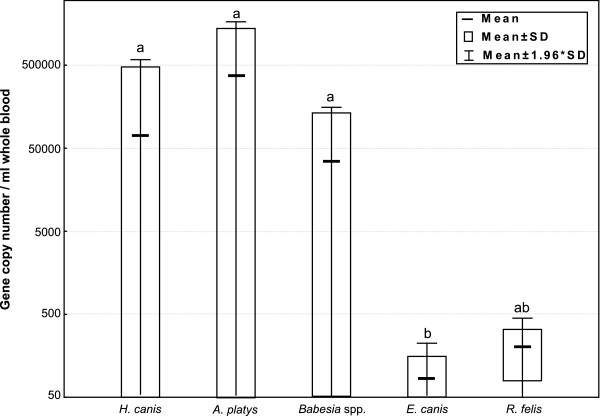
**Gene copy number of five vector-borne pathogens in canine whole blood samples.** Quantitative FRET-PCRs determined the gene copy number of five tick-borne pathogens. The rRNA copy numbers for *E. canis* (84 ± 71 /per ml whole blood; range 50 ~ 313) were significantly lower than those for *H. canis* (72,120 ± 260,148), *A. platys* (370472 ± 659438; range 651 ~ 1534409), and *B. canis* (35161 ± 61,335; range 72 ~ 199,552). The *gltA* copy number for *R. felis* (204 ± 125; range 116 and 292) was not significantly different from the rRNA copy number of the other four tick-borne agents. The Y-axis is shown as logarithmic scale, and the data were shown as average gene copy number (-), mean ± SD (□) and mean ±1.96 × SD (Ι).

The most common infectious agent we detected was *E. canis* (22/39; 56%) with an average of 84 copies of the 16S rRNA gene/ml whole blood (SD 71; range 50 ~ 313) (Figure 1). This prevalence is similar to the 47% (148/310) [6] to 34% (50/146) [8] reported from Costa Rica in the only other PCR surveys of dogs in Central America, and these findings suggest the organism is prevalent in the region. Infections with *E. canis* cause canine monocytic ehrlichiosis with 80% of dogs developing prolonged thrombocytopenia [26,27]. We were unable to carry out hematological examinations on our study dogs but, despite the high likelihood that some were thrombocytopenic, all the surgeries were uneventful and no abnormal bleeding tendencies were noted.

*E. canis* is transmitted by the *Rhipicephalus sanguineus* group which occurs worldwide [28]. There is no vaccine for *E. canis* and only tick control can prevent infections. Unfortunately, *R. sanguineus* group is notoriously difficult to control even with expensive modern acaricides and applicators. Diagnosing infections is also challenging as, although organisms can be seen in stained blood smears, microscopy is rarely successful and diagnoses are usually made with expensive serology and/or PCRs. Even when diagnoses are made, treatment failures are common in naturally infected dogs [17,29], most probably as a result of inadequate tick control and re-infection. The difficulties associated with tick control, diagnosis and treatment mean that in underprivileged areas infections with *E. canis* will be particularly difficult to control and canine monocytic ehrlichiosis can be expected to remain prevalent in these areas.

Although apparently uncommon, *E. canis* can infect people [30] and health workers in Central America should be aware of the high prevalence of ehrlichiosis in dogs and the possibility of infections in their patients, particularly those with a history of tick bites [8,9,31].

Infections with *H. canis* were also very common in our study (20/39; 51%) with an average 18S rRNA copy number of 72,120/ml whole blood (SD 260,148; range 3 ~ 1,171,720) (Figure 1). A lower infection rate was found in Costa Rica (11/146; 8%) in the only other investigation of the organism in Central America [8]. Infections have also been reported from Africa, Southwestern Asia, Southwestern and Eastern Europe and North and South America [3], indicating a worldwide distribution of the organism. This is expected as the main vector of *H. canis* is *R. sanguineus* group [32], which occurs worldwide [28]. Most infections are subclinical, but in some dogs (15%) there is high parasitemia and clinical signs including fever, weight loss, anemia and hyperglobulinemia [33]. Many dogs have concurrent infections with vector-borne agents such as *E. canis* and *Babesia* spp., and deciding each agent’s contribution to signs is difficult. Various treatments have been used but none are fully effective [34].

The predominant *Babesia* spp. in dogs are *B. gibsoni*, *B. canis*, *B. vogeli*, and *B. rossi*[35-37]. In our study we found 26% of the dogs (10/39) had DNA of *Babesia* spp. with an average 18S rRNA copy number of 370,472/ml whole blood (SD 659,438; range 72 ~ 199,552). Melting point analysis and genomic sequencing showed four of the 10 dogs to be infected with *B. gibsoni* (*T*_m_ ~67°C) and six with *B. vogeli* (*T*_m_ ~58°C). None of the dogs were infected with both species or with *B. vogeli* or *B. rossi. B. gibsoni* is found in northern Africa, southern Asia, Australia, Europe, the USA and the Caribbean [17] and ours is the first report of the organism in Central America. The organism is thought to be transmitted by fighting or by ticks, in particular *R. sanguineus* group [38]. There are no reported human infections [39] but in dogs infections usually result in acute signs including fever, pallor, splenomegaly and anorexia [39]. Dogs that recover from the acute infection generally become chronic subclinical carriers with significantly reduced platelet counts [40]. Diagnosis is by detecting organisms in stained blood smears but in chronic cases organisms are seldom seen and serology or PCR are needed [39]. As with canine ehrlichiosis, treatment of *B. gibsoni* is difficult and often the parasite is not eliminated [41].

The other *Babesia* spp. we detected, *B. vogeli*, has been reported from Central America with 8% of 146 dogs studied in Costa Rica being positive [8]. This organism is transmitted by *R. sanguineus* group, with infections being subclinical or causing only mild signs with anemia and thrombocytopenia [4]. It has also not been reported to infect people [38].

Only a few dogs (5/39, 13%) carried *A. platys* DNA with an average of 370,472 copies of the 16S rRNA gene/ml whole blood (SD 659,438; range 651 ~ 1,534,409). This is similar to the prevalence reported in Costa Rica (14/146: 10%) [8] and indicates the organism is probably widely distributed in Central America, as it is elsewhere in the world. *A. platys* is probably transmitted by *R. sanguineus* group and is the agent of infectious canine cyclic thrombocytopenia [42]. There is only a single report of an infection in a person [43]. Although infections in dogs are often subclinical, infected dogs might be thrombocytopenic. Platelet numbers might be lower with coinfections with other vector-borne agents, in particular *E. canis* and *Babesia* spp. [17,44]. Although organisms can be seen in peripheral blood smears, the most sensitive diagnosis is by serology or PCR [42]. Treatment with doxycycline has been reported to sterilize infections [45].

The least common agent we detected was *R. felis* (2/39, 5%) with *gltA* copies of 116 and 292/ml of whole blood (SD 125; range 116 ~ 292). This is an emerging pathogen principally associated with cat fleas (*Ctenocephalides felis*) and responsible for flea-borne spotted fever in people [46]. The organism occurs on all continents except Antarctica and has been reported in cat fleas from Guatemala, Costa Rica and Panama in Central America [9,47,48]. Recent evidence has indicated dogs might be reservoir hosts for the organism although infections seem to be subclinical [14]. Infections in people can cause clinical signs ,which are generally mild and consist of fever, headache, myalgia and rash [49]. In Africa, infections appear common with up to 6% of febrile patients in Kenya and Senegal reported to be PCR positive for the organism [50]. Although no clinical cases have been reported from Central America, *R. felis* appears to be widespread in the area and health workers should be aware of the possibility of flea-borne spotted fever in patients with a history of contact with fleas.

In the 80% (31/39) of dogs we studied which had DNA of a vector-borne agent, mixed infections were very common with 11 (28%) having evidence of two agents, 3 (8%) having evidence of three, and 4 (10%) of four agents (Table 1). The most common multiple infection was with *H. canis* and *E. canis* (7 dogs) with infections with *H. canis*, *E. canis*, *B. vogeli* and *A. platys* being second most common (3 dogs). There is little detailed data on mixed infections and how one agent may exacerbate [8,17,44] or ameliorate [51] the effects of another, which greatly complicated clinical diagnoses. Also it means that in many dogs multiple drug therapies are needed to cure animals and resolve hematological and biochemical abnormalities.

**Table 1:** Prevalences of vector-borne agents in dogs determined by quantitative PCRs

**Dog number**	**Tick-borne pathogens**				
	***Rickettsia***	***Hepatozoon***	***Babesia***	***Anaplasma***	***Ehrlichia***
1	-	*H. canis*	*B. vogeli*	*A. platys*	*E. canis*
2	-	-	-	-	*E. canis*
3	-	-	-	-	-
4	-	-	*B. gibsoni*	*A. platys*	*E. canis*
5	-	-	*B. gibsoni*	-	-
6	-	-	*B. vogeli*	-	*E. canis*
7	-	*H. canis*	-	-	*E. canis*
8	-	-	-	-	-
9	-	-	-	-	*E. canis*
10	-	*H. canis*	-	-	-
11	-	*H. canis*	-	-	*E. canis*
12	*R. felis*	-	-	-	*E. canis*
13	-	*H. canis*	-	-	*E. canis*
14	-	-	-	-	*E. canis*
15	-	-	-	-	-
16	-	*H. canis*	-	-	*E. canis*
17	-	-	-	-	-
18	-	-	-	-	*E. canis*
19	-	-	-	-	-
20	-	*H. canis*	*B. vogeli*	*A. platys*	*E. canis*
21	-	*H. canis*	-	*A. platys*	-
22	-	-	-	-	*E. canis*
23	-	-	-	-	-
24	-	*H. canis*	*B. vogeli*	*A. platys*	*E. canis*
25	-	*H. canis*	*B. vogeli*	-	*E. canis*
26	-	-	-	-	-
27	-	-	-	-	-
28	-	*H. canis*	-	-	*E. canis*
29	-	*H. canis*	-	-	-
30	-	*H. canis*	-	-	-
31	-	*H. canis*	*B. gibsoni*	-	-
32	-	*H. canis*	-	-	-
33	-	*H. canis*	-	-	*E. canis*
34	-	-	-	-	-
35	-	*H. canis*	*B. gibsoni*	-	*E. canis*
36	-	-	-	-	*E. canis*
37	*R. felis*	*H. canis*	*B. vogeli*	-	*E. canis*
38	-	*H. canis*	-	-	-
39	-	*H. canis*	-	-	*E. canis*

“-”denotes the absence of examined DNA in the whole blood of the designated dog.

It is particularly noteworthy that many of the pathogens we found cause thrombocytopenia in apparently healthy dogs. The infections are often difficult to diagnose without expensive serology and/or PCR, and treatment is often unsuccessful which poses major problems to local veterinarians carrying out surgeries and organizations carrying out neutering programs in underprivileged areas. Finding innovative and inexpensive methods for parasite and vector control on dogs appears to be the best way forward for controlling these vector-borne pathogens of dogs and people.

The nucleotide sequences of the PCR amplicons we obtained for the five vector-borne pathogens we detected were identical to those reported previously. The sequences of the *R. felis*, *B. gibsoni*, *B. vogeli*, *H. canis*, *A. platys* and *E. canis* amplicons were the same as those reported in GenBank for organisms from Brazil (Gene Accession #JF694092), St. Kitts (JX112784), St Kitts (JX112785), Brazil (KF692040), South Africa (KC189854) and the Czech Republic (KC479024), respectively.

Comparisons of the average gene copy numbers of the five vector-borne pathogens we studied showed the rRNA copy numbers for *E. canis* were significantly lower than those for *H. canis*, *B. canis* and *A. platys* (Figure 1; Kruskal-Wallis non-parametric ANOVA). This probably reflects the fact that *E. canis* occurs in monocytes which are in lower concentrations in the blood than neutrophils, erythrocytes and platelets in which the other organisms are found, respectively. The *gltA* copy number for *R. felis* was not significantly different from the rRNA copy number of the other four tick-borne agents.

## Conclusions

Our study has added to the scant data on canine and zoonotic vector-borne agents in Central America. We provide the first data on these organisms in dogs in Nicaragua and the first report of *B. gibsoni* in Central America. The available data from our study and the few others performed in the region suggest vector-borne diseases of dogs are common and widespread and in need of further investigation. Also, many of the pathogens can infect people and health workers need to be aware of this possibility, particularly in patients that have contact with dogs and their parasites.

## Abbreviations

HMBS: Hydroxymethylbilane synthase; VIDA: Intercultural and Definitive Adventure; R.: Rickettsia; B.: Babesia; H.: Hepatozoon; A.: Anaplasma; E.: Ehrlichia; C.: Coxiella; D.: Dirofilaria; R.: Rhipicephalus.

## Competing interests

The authors have declared no conflict of interests.

## Authors’ contributions

LW, PK, BK and CW designed the experiment, monitored the study and interpreted the results. LW, KA, JZ and HSE performed the experiment. All authors read and approved the final version of the manuscript.

## References

[B1] Groves MG Dennis GL Amyx HL Huxsoll DL Transmission of *Ehrlichia canis* to dogs by ticks (*Rhipicephalus sanguineus*) Am J Vet Res 1975 36 937 940 1147359

[B2] Harrus S Waner T Diagnosis of canine monocytotropic ehrlichiosis (*Ehrlichia canis*): an overview Vet J 2011 187 292 296 10.1016/j.tvjl.2010.02.001 20226700

[B3] O’Dwyer LH Brazilian canine hepatozoonosis Rev Bras Parasitol Vet 2011 20 181 193 10.1590/S1984-29612011000300002 21961746

[B4] Solano-Gallego L Baneth G Babesiosis in dogs and cats-expanding parasitological and clinical spectra Vet Parasitol 2011 181 48 60 10.1016/j.vetpar.2011.04.023 21571435

[B5] Cardoso Cardoso L Mendão C Madeira De Carvalho L Prevalence of *Dirofilaria immitis*, *Ehrlichia canis*, *Borrelia burgdorferi* sensu lato, *Anaplasma* spp. and *Leishmania infantum* in apparently healthy and CVBD-suspect dogs in Portugal--a national serological study Parasit Vectors 2012 5 62 10.1186/1756-3305-5-62 22452990 PMC3353209

[B6] Romero LE Meneses AI Salazar L Jiménez M Romero JJ Aguiar DM Labruna MB Dolz G First isolation and molecular characterization of *Ehrlichia canis* in Costa Rica, Central America Res Vet Sci 2011 91 95 97 10.1016/j.rvsc.2010.07.021 20723954

[B7] Scorza AV Duncan C Miles L Lappin MR Prevalence of selected zoonotic and vector-borne agents in dogs and cats in Costa Rica Vet Parasitol 2011 183 178 183 10.1016/j.vetpar.2011.06.025 21846585

[B8] Rojas A Rojas D Montenegro V Gutiérrez R Yasur-Landau D Baneth G Vector-borne pathogens in dogs from Costa Rica First molecular description of *Babesia vogeli* and *Hepatozoon canis* infections with a high prevalence of monocytic ehrlichiosis and the manifestations of co-infection Vet Parasitol 2014 199 121 128 10.1016/j.vetpar.2013.10.027 24315693

[B9] Bermúdez CS Zaldívar AY Spolidorio MG Moraes-Filho J Miranda RJ Caballero CM Mendoza Y Labruna MB Rickettsial infection in domestic mammals and their ectoparasites in El Valle de Antón, Coclé, Panamá Vet Parasitol 2011 177 134 138 10.1016/j.vetpar.2010.11.020 21144663

[B10] Pineda V Saldaña A Monfante I Santamaría A Gottdenker NL Yabsley MJ Rapoport G Calzada JE Prevalence of trypanosome infections in dogs from Chagas disease endemic regions in Panama, Central America Vet Parasitol 2011 178 360 363 10.1016/j.vetpar.2010.12.043 21273002

[B11] Herrer A Christensen HA Natural cutaneous leishmaniasis among dogs in Panama Am J Trop Med Hyg 1976 25 59 63 943961 10.4269/ajtmh.1976.25.59

[B12] Ryan PR Arana BA Ryan JR Wirtz RA Wortmann GW Rizzo NR The domestic dog, a potential reservoir for *Leishmania* in the Peten region of Guatemala Vet Parasitol 2003 115 1 7 10.1016/S0304-4017(03)00158-4 12860062

[B13] Zhang J Wei L Kelly P Freeman M Jaegerson K Gong J Xu B Pan Z Xu C Wang C Detection of *Salmonella* spp. Using a Generic and Differential FRET-PCR PLoS One 2013 8 e76053 10.1371/journal.pone.0076053 24146814 PMC3797804

[B14] Hii SF Abdad MY Kopp SR Stenos J Rees RL Traub RJ Seroprevalence and risk factors for *Rickettsia felis* exposure in dogs from Southeast Queensland and the Northern Territory, Australia Parasit Vectors 2013 6 159 10.1186/1756-3305-6-159 23731951 PMC3679791

[B15] Wang C Ahluwalia SK Li Y Gao D Poudel A Chowdhury E Boudreaux MK Kaltenboeck B Frequency and therapy monitoring of canine *Babesia* spp. infection by high-resolution melting curve quantitative FRET-PCR Vet Parasitol 2010 168 11 18 10.1016/j.vetpar.2009.10.015 19931290

[B16] Li Y Wang C Allen KE Little SE Ahluwalia SK Gao D Macintire DK Blagburn BL Kaltenboeck B Diagnosis of canine *Hepatozoon* spp. infection by quantitative PCR Vet Parasitol 2008 157 50 58 10.1016/j.vetpar.2008.06.027 18774228

[B17] Kelly PJ Xu C Lucas H Loftis A Abete J Zeoli F Stevens A Jaegersen K Ackerson K Gessner A Kaltenboeck B Wang C Ehrlichiosis, babesiosis, anaplasmosis and hepatozoonosis in dogs from St. Kitts, West Indies PLoS One 2013 8 e53450 10.1371/journal.pone.0053450 23335965 PMC3546050

[B18] Berri M Rekiki A Boumedine KS Rodolakis A Simultaneous differential detection of *Chlamydophila abortus,*Chlamydophila pecorum and Coxiella burnetii from aborted ruminant’s clinical samples using multiplex PCR BMC Microbiol 2009 9 130 10.1186/1471-2180-9-130 19570194 PMC2725139

[B19] Wang C Mount J Butler J Gao D Jung E Blagburn BL Kaltenboeck B Real-time PCR of the mammalian hydroxymethylbilane synthase (HMBS) gene for analysis of flea (*Ctenocephalides felis*) feeding patterns on dogs Parasit Vectors 2012 5 4 10.1186/1756-3305-5-4 22214496 PMC3275464

[B20] McCall JW Genchi C Kramer LH Guerrero J Venco L Heartworm disease in animals and humans Adv Parasitol 2008 66 193 285 18486691 10.1016/S0065-308X(08)00204-2

[B21] Levy JK Lappin MR Glaser AL Birkenheuer AJ Anderson TC Edinboro CH Prevalence of infectious diseases in cats and dogs rescued following Hurricane Katrina J Am Vet Med Assoc 2011 238 311 317 10.2460/javma.238.3.311 21281213

[B22] Cutler SJ Bouzid M Cutler RR Q fever J Infect 2007 54 313 318 10.1016/j.jinf.2006.10.048 17147957

[B23] Marrie TJ Durant H Williams JC Exposure to parturient cats: a risk factor for acquisition of Q fever in Maritime Canada J Infect Dis 1988 158 101 108 10.1093/infdis/158.1.101 3392409

[B24] Buhariwalla F Cann B Marrie TJ A dog-related outbreak of Q fever Clin Infect Dis 1996 23 753 755 10.1093/clinids/23.4.753 8909839

[B25] Hornok S Dénes B Meli ML Tánczos B Fekete L Gyuranecz M de la Fuente J De Mera IG Farkas R Hofmann-Lehmann R Non-pet dogs as sentinels and potential synanthropic reservoirs of tick-borne and zoonotic bacteria Vet Microbiol 2013 167 700 703 10.1016/j.vetmic.2013.08.011 24021884

[B26] Kelly PJ Canine ehrlichioses: an update J S Afr Vet Assoc 2000 71 77 86 11030356 10.4102/jsava.v71i2.684

[B27] Shaw SE Day MJ Birtles RJ Breitschwerdt EB Tick-borne infectious diseases of dogs Trends Parasitol 2001 17 74 80 10.1016/S1471-4922(00)01856-0 11228013

[B28] Dantas-Torres F The brown dog tick, *Rhipicephalus sanguineus* (Latreille, 1806) (Acari: Ixodidae): from taxonomy to control Vet Parasitol 2008 152 173 185 10.1016/j.vetpar.2007.12.030 18280045

[B29] Bartsch RC Greene RT Post-therapy antibody titers in dogs with ehrlichiosis: follow-up study on 68 patients treated primarily with tetracycline and/or doxycycline J Vet Intern Med 1996 10 271 274 10.1111/j.1939-1676.1996.tb02061.x 8819054

[B30] Perez M Bodor M Zhang C Xiong Q Rikihisa Y Human infection with Ehrlichia canis accompanied by clinical signs in Venezuela Ann N Y Acad Sci 2006 1078 110 117 10.1196/annals.1374.016 17114689

[B31] Troyo A Calderón-Arguedas O Alvarado G Vargas-Castro LE Avendaño A Ectoparasites of dogs in home environments on the Caribbean slope of Costa Rica Rev Bras Parasitol Vet 2012 21 179 183 10.1590/S1984-29612012000200021 22832763

[B32] Baneth G Samish M Shkap V Life cycle of *Hepatozoon canis* (Apicomplexa: Adeleorina: Hepatozoidae) in the tick *Rhipicephalus sanguineus* and domestic dog (*Canis familiaris*) J Parasitol 2007 93 283 299 10.1645/GE-494R.1 17539411

[B33] Baneth G Perspectives on canine and feline hepatozoonosis Vet Parasitol 2011 181 3 11 10.1016/j.vetpar.2011.04.015 21620568

[B34] Pasa S Voyvoda H Karagenc T Atasoy A Gazyagci S Failure of combination therapy with imidocarb dipropionate and toltrazuril to clear *Hepatozoon canis* infection in dogs Parasitol Res 2011 109 919 926 10.1007/s00436-011-2334-3 21472405

[B35] Homer MJ Aguilar-Delfin I Telford SR 3rd Krause PJ Persing DH Babesiosis Clin Microbiol Rev 2000 13 451 469 10.1128/CMR.13.3.451-469.2000 10885987 PMC88943

[B36] Irwin PJ Canine babesiosis: from molecular taxonomy to control Parasit Vectors 2009 2 Suppl 1 S4 10.1186/1756-3305-2-S1-S4 19426443 PMC2679396

[B37] Taboada J Lobetti R Greene CE Babesiosis Infectious Diseases of the Dog and Cat 2006 Missouri: Saunders Elsevier 722 736

[B38] Esch KJ Petersen CA Transmission and epidemiology of zoonotic protozoal diseases of companion animals Clin Microbiol Rev 2013 26 58 85 10.1128/CMR.00067-12 23297259 PMC3553666

[B39] Ayoob AL Hackner SG Prittie J clinical management of canine babesiosis J Vet Emerg Crit Care (San Antonio) 2010 20 77 89 10.1111/j.1476-4431.2009.00489.x 20230437

[B40] Matsuu A Kawabe A Koshida Y Ikadai H Okano S Higuchi S Incidence of canine *Babesia gibsoni* infection and subclinical infection among Tosa dogs in Aomori Prefecture Japan J Vet Med Sci 2004 66 893 897 10.1292/jvms.66.893 15353837

[B41] Iguchi A Matsuu A Fujii Y Ikadai H Hikasa Y The in vitro interactions and in vivo efficacy of atovaquone and proguanil against *Babesia gibsoni* infection in dogs Vet Parasitol 2013 197 527 533 10.1016/j.vetpar.2013.06.006 24075418

[B42] Alleman AR Wamsley HL An update on anaplasmosis in dogs Vet Med 2008 103 212 220

[B43] Maggi RG Mascarelli PE Havenga LN Naidoo V Breitschwerdt EB Co-infection with *Anaplasma platys,* Bartonella henselae and Candidatus Mycoplasma haematoparvum in a veterinarian Parasit Vectors 2013 6 103 10.1186/1756-3305-6-103 23587235 PMC3637287

[B44] Brown GK Canfield PJ Dunstan RH Roberts TK Martin AR Brown CS Irving R Detection of *Anaplasma platys* and *Babesia canis vogeli* and their impact on platelet numbers in free-roaming dogs associated with remote Aboriginal communities in Australia Aust Vet J 2006 84 321 325 10.1111/j.1751-0813.2006.00029.x 16958629

[B45] Chang WL Su WL Pan MJ Two-step PCR in the evaluation of antibiotic treatment for *Ehrlichia platys* infection J Vet Med Sci 1997 59 849 851 10.1292/jvms.59.849 9342716

[B46] Abdad M Stenos J Graves S *Rickettsia felis*, an emerging flea-transmitted human pathogen Emerg Health Threats J 2011 4 7168 24149035 10.3402/ehtj.v4i0.7168 PMC3168219

[B47] Hun L Troyo A Taylor L Barbieri AM Labruna MB First report of the isolation and molecular characterization of *Rickettsia amblyommii* and *Rickettsia felis* in Central America Vector Borne Zoonotic Dis 2011 11 1395 1397 10.1089/vbz.2011.0641 21612539

[B48] Troyo A Álvarez D Taylor L Abdalla G Calderón-Arguedas Ó Zambrano ML Dasch GA Lindblade K Hun L Eremeeva ME Estévez A *Rickettsia felis* in *Ctenocephalides felis* from Guatemala and Costa Rica Am J Trop Med Hyg 2012 86 1054 1056 10.4269/ajtmh.2012.11-0742 22665618 PMC3366522

[B49] Pérez-Osorio CE Zavala-Velázquez JE Arias León JJ Zavala-Castro JE *Rickettsia felis* as emergent global threat for humans Emerg Infect Dis 2008 14 1019 1023 10.3201/eid1407.071656 18598619 PMC2600327

[B50] Richards AL Ju J Sylvia O Ryan D Khalif A Abdile A Sharif SK Feikin DR Breiman RF Kariuki Njenga M Human Infection with *Rickettsia felis*, Kenya Emerg Infect Dis 2010 16 1081 1086 10.3201/eid1607.091885 20587178 PMC3321909

[B51] Matthewman LA Kelly PJ Bobade PA Tagwira M Mason PR Majok A Brouqui P Raoult D Infections with *Babesia canis* and *Ehrlichia canis* in dogs in Zimbabwe Vet Rec 1993 133 344 346 10.1136/vr.133.14.344 8236678

